# The influence of reward in the Simon task: Differences and similarities to the Stroop and Eriksen flanker tasks

**DOI:** 10.3758/s13414-022-02563-7

**Published:** 2022-10-31

**Authors:** Victor Mittelstädt, Rolf Ulrich, Julia König, Katharina Hofbauer, Ian Grant Mackenzie

**Affiliations:** Department of Psychology, University of Tübingen, Schleichstraße 4, 72076 Tübingen, Germany

**Keywords:** Simon effect, Eriksen flanker effect, Stroop, Effect, Conflict task, Cognitive control, Reward, Delta plots

## Abstract

Previous studies have suggested that performance-contingent reward can modulate cognitive control by biasing irrelevant location-response associations in the Simon task. However, the influence of reward in the case of irrelevant words (Stroop task) or irrelevant flankers (Eriksen Flanker task) remains unclear. Across two preregistered experiments, the present study investigated the influence of reward on conflict processing with different types of distractors. Conflict effects on mean reaction time (RT) were reduced in the Simon task (Experiments 1 and 2) when incongruent versus congruent trials were rewarded, and this modulating effect of reward on conflict processing was also observed in the Eriksen flanker task (Experiment 2), but not in the Stroop task (Experiment 1). We propose that cognitive control adjustments to distractor-specific reward contingencies can be generalized across distractor types producing both perceptual-related (Flanker task) and motor-related (Simon task) conflict, but, if any, to a limited degree when distractors produce additional higher-level task conflict (Stroop task). In addition, distributional RT analyses (delta plots) revealed that rewarded distractor-response associations modulate cognitive control not only via biasing the strength (Simon and Eriksen tasks) but also the time-course of suppressing distractor processing (Eriksen task). Overall, the present study dissociated distractor-general and distractor-specific effects of reward on cognitive control.

We are constantly required to select and process task-relevant information in environments overloaded with distracting information. Thus, adaptive goal-directed behavior requires sophisticated control mechanisms that help to flexibly shield target processing from potentially harmful distracting information (e.g., Verbruggen et al., 2014; Shiffrin & Schneider, 1977; Braver, 2012; Braem & Egner, 2018). To uncover these mechanisms in the laboratory, researchers often study behavior using a variety of so-called conflict tasks (e.g., Stroop, 1935; Eriksen & Eriksen, 1974; Simon & Rudell, 1967). In these tasks, participants are required to select a response based on a target-dimension while ignoring the non-target dimension (e.g., location in the Simon task or word meaning in the Stroop task, e.g., Chen et al., 2020; Prével et al., 2021). Conflict effects indicate that distractors affect target processing because responses are typically faster and less error prone when the response associated with the two dimensions match (congruent trials) as compared to mismatch (incongruent trials). The size of conflict effects is often modulated by common factors suggesting that conflict processing with different types of distractors can be conceptualized within single processing architectures (e.g., dual-route models Ulrich et al., 2015). However, some findings also imply that at least partially distractor-specific control mechanisms may operate across conflict tasks (e.g., Mackenzie et al., 2022; Kornblum et al., 1990; Hommel, 2011; Egner, 2007). Clearly, the question how different types of distractors interact with target processing is central to our understanding of cognitive control.

In the present study, we aim to provide some further insights into the role of potential distractor-specific versus distractor-general processes by investigating to what extent rewarding congruent versus incongruent trials may differentially affect conflict processing in three prominent conflict tasks—the Simon task, the Stroop task and the Eriksen flanker task. As is elaborated next, there is strong evidence that reward strengthens congruent (as opposed to incongruent) distractor-response associations and increases the Simon effect (Chen et al., 2020). However, there are some hints that this manipulation is less (or not) effective in modulating the Stroop effect (Prével et al., 2021), and it is unclear to what extent reward influences the Eriksen flanker effect.

## Reward in conflict tasks

In the present study, we investigate the influence of performance-contingent reward on conflict processing within the Simon, Stroop, and Eriksen flanker tasks (e.g., Janczyk & Leuthold, 2017; Servant et al., 2016; Steinhauser & Hübner, 2009). Specifically, in these conflict tasks, participants are instructed to respond with left versus right key presses a) to the task-relevant feature (e.g., color) of a lateralized stimulus (= Simon task) b) to the ink color of colored word stimuli (e.g., RED written with green font) (= Stroop task) and c) to the color of a centrally positioned circle flanked on each side by colored circles (= Eriksen flanker task). In all of these conflict tasks, it is essentially assumed that activation produced by distractor-based information (i.e., location, word meaning, flankers) superimpose with activation produced by target-based information (here colors) during decision-making thereby improving (congruent) or impairing (incongruent) task performance in terms of reaction time (RT) and error rates (Ulrich et al., 2015; Eimer et al., 1995; Ridderinkhof et al., 1995; Rey-Mermet et al., 2021; Hübner et al., 2010; Wühr & Heuer, 2018; Miller & Schwarz, 2022; Stürmer et al., 2002).

The effects of reward on cognitive control are typically investigated by comparing the size of conflict effects in conditions where reward can versus cannot be obtained for good performance (Krebs & Woldorff, 2017). Surprisingly, even though main effects of reward (i.e., faster RT in rewarded compared to non-rewarded conditions) were present in previous studies, findings concerning the modulation of conflict effects by reward are mixed. For example, some studies have used cues in advance of trials or blocks in order to signal prospective performance-contingent reward (e.g., Yamaguchi & Nishimura, 2019; Padmala & Pessoa, 2011; Bundt et al., 2021; Frömer et al., 2021). Because participants know in advance of target onset that they can obtain reward, it is generally assumed that participants proactively bias motivation-related control processes in anticipation of potential reward. While this manipulation led to reduced conflict effects in reward compared to no-reward conditions in the Eriksen flanker task (Yamaguchi & Nishimura, 2019), this was not the case in the Simon task (Bundt et al., 2016), and the findings within the Stroop task itself are mixed (Padmala & Pessoa, 2011; Bundt et al., 2021). Furthermore, some other studies associated prospective performance-contingent reward with specific target features (e.g., specific color or letter Wang et al., 2019; Krebs et al., 2010). Here, it is usually assumed that reward affects learning-related control processes (e.g., by strengthening the reward-specific target-response links) and participants adjust their processing during a trial as a function of rewarding target features. This manipulation yielded reduced conflict effects with reward compared to no-reward target features in the Stroop task (Krebs et al., 2010), but produced exactly the opposite pattern in the Simon task (Wang et al., 2019). Assuming that these different effects across conflict tasks are not the result of some experimental particularities, reconciling these findings may imply that the reward-induced control processes may at least partially differentially affect conflict resolution with different types of distractors (e.g., Kornblum et al., 1990).

Critically, conflict task-specific reward sensitivity may also play a role when comparing the findings from two recent studies where performance-contingent reward was directly associated to conflict processing. First, in a Simon task study by (Chen et al., 2020) participants were either exclusively rewarded following congruent trials (RC group) or following incongruent trials (RI group). Across two experiments, the Simon effects were considerably reduced (and even reversed) in the RI as compared to the RC group. The authors concluded that reward can modulate cognitive control by biasing (irrelevant) distractor-response associations. Second, (Prével et al., 2021) essentially applied the same approach in a Stroop task across three experiments. Interestingly, the Stroop effect was only significantly reduced in Experiment 3 but not in Experiments 1 and 2.^1^ Furthermore, the changes in the Stroop effect were only observed for the specific rewarded trials (learning trials) and did not transfer to unrewarded congruent versus incongruent trials (neutral trials). In contrast, (Chen et al., 2020) observed that the reward-based modulation of the Simon effect was present for both learning and neutral trials. Thus, it seems that rewarding distractor-response association is much more effective in the Simon than in the Stroop task. On the one hand, this implies additional assumptions about differences between the two tasks. For example, perhaps the distracting word features in the Stroop task might produce somewhat different conflict than the distracting location in the Simon task (e.g., stimulus-related versus response-related conflict, cf. Kornblum et al., 1990). On the other hand, the two studies differed in several other respects (e.g., within versus between-subject design, reward structures etc.) and thus potential different effects can be solely explained by subtle methodological differences.^2^

In order to shed more light on potential distractor-specific versus distractor-general control mechanisms, we directly investigated whether different types of distractors (and/or conflicts) are indeed potentially differentially sensitive to reward or not when controlling for methodological artifacts. More precisely, across two preregistered experiments, we examined whether rewarding incongruent versus congruent trials modulate the size of conflict effects a) in the Simon versus Stroop task (Experiment 1) and b) in the Simon versus Eriksen flanker task (Experiment 2).

Because examining conflict effects across time could allow insights into the underlying processing mechanisms that are not available when looking only at mean RTs (e.g., Mittelstädt & Miller, 2020; Gade et al., 2020; Wiegand & Wascher, 2005; Van Zandt, 2002), we will also conduct more fine-grained RT analyses at a distributional level. Specifically, we will compare the condition-specific so-called delta plots which illustrate the size of conflict effects as a function of response speed (e.g., Burle et al., 2013; De Jong et al., 1994; Luo & Proctor, 2020; Ridderinkhof, 2002; Hübner & Töbel, 2019; Schwarz & Miller, 2012). The slope of delta plots usually markedly differs across conflict tasks (i.e., rather decreasing slopes in the Simon task and rather increasing slopes in the Stroop and Eriksen flanker tasks, cf. Pratte et al., 2010; Mittelstädt et al., 2022; Ellinghaus et al., 2017; Kinoshita et al., 2017). These different slopes may reflect some differences between tasks (e.g., distractor-speed and/or early versus late locus of conflict) and observing a distinct distributional pattern in the present study could point to the presence of distractor-specific processes. Furthermore, the slope of delta plots may also reflect the time-course of cognitive control (e.g., Ridderinkhof, 2002). Thus, observing differences in slopes as a function of the reward manipulation could indicate that rewarding distractor-response association may modulate cognitive control by affecting the timing of distractor suppression.

## Experiment 1

In the first experiment, we investigated whether and how reward contingencies influenced the size of conflict effects in the Simon and Stroop task. Thus, all participants were required to respond to the same target feature (i.e., color) in Simon task and Stroop task blocks, but half of the participants were selectively rewarded for congruent trials (RC group) and the other half of participants were selectively rewarded for incongruent trials. Note that the basic method and procedure (e.g., reward manipulation) were modeled closely after those used by (Chen et al., 2020) where strong reward × congruency modulations were observed.

### Method

#### Participants

60 people were tested online (41 female, 50 right-handed), ranging in age from 18 to 74 years (*M* = 27.55). They were recruited via advertisements on the campus of the University of Tübingen, social media and internal departmental e-mail lists. Data of one participant were excluded due to high mean error rates (> 25%). Note that in this and the other experiment, the results were similar when including the data of excluded participants. Furthermore, in this and the other experiment, all participants gave informed consent, were tested in a single session lasting approximately 40 min, and received course credits for participation.

#### Apparatus and stimuli

**Fig. 1:**
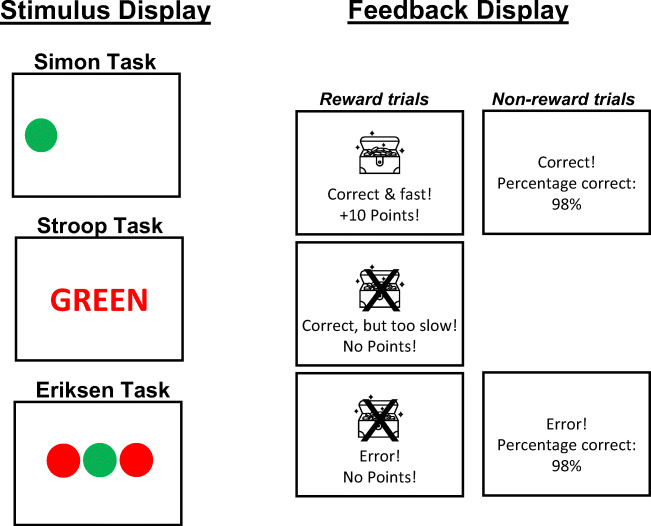
Sketch of the stimulus and feedback display (not to scale) Experiment 1 (Simon, Stroop) and Experiment 2 (Simon, Eriksen). See text for more details

The experiment was conducted online using the JavaScript library jsPsych (e.g., De Leeuw, 2015). All visual stimuli were presented on a grey background. For each participants, two colors (red: RGB[255,0,0]; green: RGB[0,255,0]) were randomly assigned to left- and right target responses. In Simon task blocks, a colored circle appeared to the left or right of the center of the screen (see Fig. 1). In the Stroop task blocks, a colored word (e.g., the German word for GREEN written in red font) was presented on the center of the screen. Responses were key presses with the left and right index fingers on the “Q” and “P” keys of a QWERTZ computer keyboard.

#### Procedure

Each participant was tested in ten blocks of 80 trials per block. Task (Simon versus Stroop) was manipulated within subjects and were held constant within a block. Half of the participants were tested in the Simon task for the first five blocks and in the Stroop task for the last five blocks, whereas this order was reversed for the other half of participants. Reward (RC group versus RI group) was manipulated between subjects. Participants were explicitly informed about the reward manipulation in advance of each block in hope to potentially maximize the reward effect. Specifically, participants were informed that they could receive points for fast and correct responses when target location and response location (Simon task) or ink color and word meaning (Stroop task) match/mismatch. We adopted the same reward scheme as (Chen et al., 2020). Thus, similar to (Chen et al., 2020) (p. 1588), reward in reward-related trials was only provided to correct responses which had an RT less than or equal to the individual mean RT of all previous correct trials of that condition. Furthermore, similar to (Chen et al., 2020), participants were told that the number of total points earned in reward trials will be multiplied with the percentage of correct trials in no reward trials in order to avoid a complete guessing strategy in no-reward trials.^3^ Finally, to further increase motivation, they were told in advance of the first block that the ten participants with the highest number of points would receive an additional 10 Euro voucher.

At the beginning of each trial the fixation cross appeared on the screen for 400 ms. Then, a colored circle was presented to the left or right side of the screen (i.e., Simon task) or a colored word was presented on the center of the screen (i.e., Stroop task). The stimulus remained on the screen until participants responded. After each response, feedback was displayed depending on whether it was a reward or non-reward trial (see Fig. 1). For reward trials, feedback indicated whether the response was 1) “correct & fast! + 10 points!” 2) “correct but too slow! No points!” or 3) “Error! No points!”. As mentioned above, “correct but too slow” responses were determined based on the adaptive response deadline in reward trials. For non-reward trials, feedback indicated whether the response was 1) “correct” or 2) “Error!” and the overall percentage of correct trials in non-reward trials. Feedback for both reward and non-reward trials was displayed for 1.5 s when the response was correct or for 2.5 s when the response was an error to further encourage correct responses across all conditions.

#### Data preparation and design

For both percentage error (PE) and reaction time (RT) analyses, the first practice block from each task type was excluded and we also discarded trials with RTs less than 150 ms as anticipations (< 0.1%) and larger than 3000 ms as outliers (< 0.1%). For RT analyses, we additionally excluded error trials (8%). As preregistered, for the central analyses on mean RTs and mean PEs we computed three mixed ANOVAs. Specifically, we first performed two 2 × 2 mixed ANOVAs with the within-subject factors of congruency (congruent, incongruent) and the between-subject factor of reward (RC, RI) separately for each task. In order to more directly compare the effects in the two tasks, we performed a 2 × 2 × 2 mixed ANOVA for which we additionally included the within-subject factors of task (Simon, Stroop).

Furthermore, we also compared the congruency effects for each condition via distributional RT analyses (i.e., delta plots). Specifically, we created 9 RT percentiles (i.e., 10%, 20%,...) separately for each participant within each of the four conditions (i.e., congruent/incongruent × Simon/ Stroop). To compare the corresponding delta plots, we computed for each participant the slope of the delta plot in each condition using a linear regression (e.g., Pratte et al., 2010; Mittelstädt & Miller, 2020; 2018; Mackenzie et al., 2022; Ellinghaus & Miller, 2018; Hübner & Töbel, 2019; Gade et al., 2020) using the R-package DMCfun (Mackenzie & Dudschig, 2021). We then conducted a mixed ANOVA with the within-subject factor of task and the between-subject factor of reward on the mean slopes (with follow-up pairwise comparison within each task). While some statistical assumptions are potentially violated when fitting regression lines to delta plots, the comparison with other methods via simulations indicated that estimating the slope based on linear regressions yielded the best results (see Pratte et al., 2010). Indeed, the corresponding slope pattern nicely fit with the visual inspection that one makes when looking at the delta plots. Nevertheless, we explored the stability of the delta plot results using slightly different procedures. First, we checked whether the results held true when omitting the first four bins to improve the linear fit (some of the observed delta plots were increasing for the first four bins and then rather stable or decreasing). Second, we checked whether the results held true when using different numbers of percentiles to create the delta plots (i.e., 5, 7, 11). In both cases, the results were very similar to the reported ones.

### Results and discussion

#### Reaction times (RTs)

**Fig. 2:**
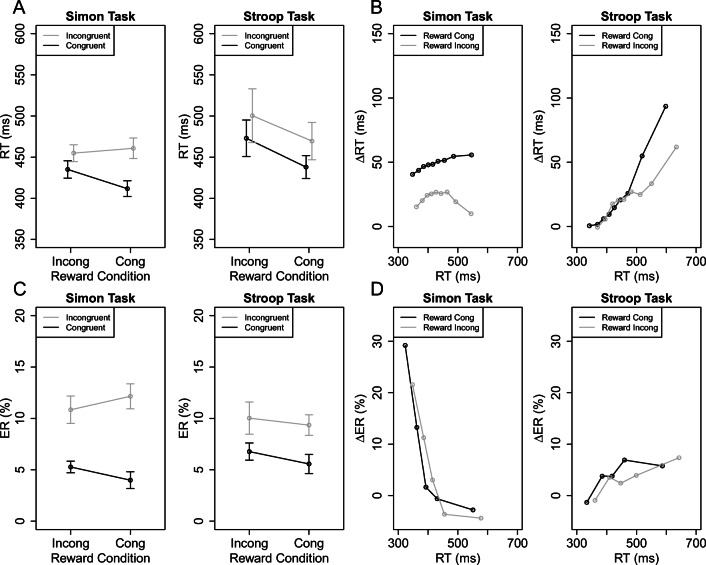
A. Mean reaction time (RT) as a function of reward (RC group, RI group) and congruency (congruent, incongruent) separately for the Simon and Stroop tasks in Experiment 1. B. Delta plots showing incongruent minus congruent differences in mean RT within each of 9 RT percentiles, plotted against the percentiles average RTs, separately for each task condition and participant. C. Mean percentage error (PE) as a function of reward group and congruency separately for the Simon and Stroop tasks. D. Delta plots showing incongruent minus congruent differences in mean PE within each of 5 RT quantiles, plotted against the quantile mean RTs, separately for each task condition and participant. The error bars in A and C indicate 1 *SE* (standard error) of the corresponding means

Figure 2A shows the mean RTs as a function of reward (RC group, RI group) and congruency (congruent, incongruent) separately for the Simon and Stroop task. The 2 × 2 Simon-ANOVA revealed a significant main effect of congruency, reflecting larger mean RT in incongruent compared to congruent trials (458-424 = 34 ms), *F*(1, 57) = 58.70, *p* < .001, ${\eta ^{2}_{p}}$ = .51. Critically, there was also a significant interaction, indicating that the Simon effect was larger in the RC than the RI group (49 ms versus 20 ms). *F*(1, 57) = 10.71, *p* = .002, ${\eta ^{2}_{p}}$ = .16. The 2 × 2 Stroop-ANOVA also revealed a significant main effect of congruency (485-456 = 29 ms), *F*(1, 57) = 13.75, *p* < .001, ${\eta ^{2}_{p}}$ = .19. However, there was no significant interaction, suggesting that there was no evidence for a larger Stroop effect in the RC than the RI group (32 ms versus 27 ms), *F*(1, 57) = 0.07, *p* = .796, ${\eta ^{2}_{p}} <$.01. The overall 2 × 2 × 2 ANOVA only revealed significant main effects of congruency, *F*(1, 57) = 40.60, *p* < .001, ${\eta ^{2}_{p}}$ = .42, and task, *F*(1, 57) = 5.21, *p* = .026, ${\eta ^{2}_{p}}$ = .08. RTs were generally slightly shorter in the Simon than Stroop task (441 ms versus 470 ms). The three-way interaction was not significant, *F*(1, 57) = 2.37, *p* = .129, ${\eta ^{2}_{p}}$ = .04. Exploratory analysis revealed, however, that there was a significant three-way interaction when additionally excluding the second block within each task condition, *F*(1, 57) = 4.50, *p* = .038, ${\eta ^{2}_{p}}$ = .07.^4^ Thus, using a Simon-Stroop-reward paradigm, rewarding incongruent versus congruent trials can reduce the mean Simon effect, but there was no evidence for a reward-specific modulation of the mean Stroop effect. This suggests that reward can differentially influence task performance with different sources of distracting information (i.e., irrelevant location versus word-meaning).^5^

Figure 2B shows the corresponding delta plots. For the 2 × 2 ANOVA with the factors task and reward on mean slopes, there was only a significant main effect of task, *F*(1, 57) = 11.10, *p* = .002, ${\eta ^{2}_{p}}$ = .16 (with all other *p* s > .112 and all other ${\eta ^{2}_{p}}$s < .04). The Stroop delta plots were generally more strongly increasing than the Simon delta plots (slopes of 0.19 and 0.03, respectively). The slopes were generally less increasing in the RI than in the RC group for both Stroop (0.15 versus 0.23) and Simon tasks (0.07 versus. -0.01), but the corresponding pairwise comparison were not significant (with *p* = .317 and *p* = .173).

As can be seen in Fig. 2B, the Stroop effect in the RC group was descriptively equal (or if anything slightly smaller) to that observed in the RI group across the first seven percentiles (i.e, RTs < 500 ms), but larger across the last percentiles (i.e, RTs > 500 ms). Exploratory analyses, however, revealed that there were no significant difference when comparing the RC versus RI Stroop effects separately at each percentile with independent *t*-tests (all *p* s >.405). Thus, there was no evidence for a reward-specific modulation of the Stroop effect even for overall larger Stroop effects. In contrast, the Simon effects were consistently less in the RI group compared to the RC group across the whole RT distribution and the differences were significant at each percentile (all *p* s <.016). Thus, the reward-specific modulation of the Simon effect was exclusively reflected in an offset of delta plots considering that there was no evidence for slope differences.^6^

#### Percentage errors (PEs)

Figure 2C shows the mean PEs as a function of reward and congruency separately for the Simon and Stroop task. The 2 × 2 Simon-ANOVA revealed only a significant main effect of congruency, reflecting larger mean PEs in incongruent compared to congruent trials (11.48% – 4.64% = 6.83%). *F*(1, 57) = 66.93, *p* < .001, ${\eta ^{2}_{p}}$ = .54, (with all other *p* s > .127 and all other ${\eta ^{2}_{p}}$s < .04). Similarly, the 2 × 2 Stroop-ANOVA yielded only a significant main effect of congruency (9.69% – 6.18% = 3.51%). *F*(1, 57) = 16.75, *p* < .001, ${\eta ^{2}_{p}}$ = .23, (with all other *p* s > .486 and all other ${\eta ^{2}_{p}}$s < .01). The overall 2 × 2 × 2 ANOVA revealed a significant main effect of congruency, *F*(1, 57) = 72.33, *p* < .001, ${\eta ^{2}_{p}}$ = .56, which was further modulated by task, *F*(1, 57) = 7.98, *p* = .006, ${\eta ^{2}_{p}}$ = .12. As can be seen in Fig. 2D, the congruency effects in PEs generally decreased with slower responses for all conditions in the Simon task, but not the Stroop task.

## Experiment 2

The first experiment demonstrated that rewarding incongruent versus congruent trials can reduce the size of conflict effects in the Simon task, but not the Stroop task. This may indicate that some conflict task-specific mechanisms operate in the Stroop than Simon task that are somehow less (or not) sensitive to the reward manipulation. For example, the Stroop versus Simon effects might arise during stimulus-related versus response-related stages of processing. Furthermore, the word-based distractors in the Stroop task may be processed slower than the location-based distractors in the Simon task, which in turn make it somehow more difficult to reactively adjust processing strategies. Indeed, the distinct delta plot pattern reinforces the idea of conflict-task specific differences (e.g., locus of interference, speed of distractor processing). In the second experiment, we aimed to see whether a similar pattern would emerge when replacing the Stroop task with the Eriksen flanker task, which also differs from the Simon task in several respects.

### Method

#### Participants

Another sample of 60 participants from the same participant pool were tested online (42 female, 52 right-handed), ranging in age from 18 to 65 years (*M* = 22.27). The data of two participants were excluded due to high mean error rates (> 25%).

#### Apparatus, stimuli and procedure

The apparatus, stimuli and procedure were the same as in Experiment 1 except for replacing the Stroop task with an Eriksen flanker task. In the Eriksen task blocks, the colored target circle was centrally presented and two colored circle appeared on each side of the target.

#### Data preparation and design

We followed the same data preparation procedure and design as in Experiment 2. For both PE and RT analyses, RTs less than 150 ms (< 0.1%) and larger than 3000 ms (< 0.1%) were excluded and for RT analyses, we additionally excluded error trials (8.2%).

#### Reaction times (RTs)

**Fig. 3:**
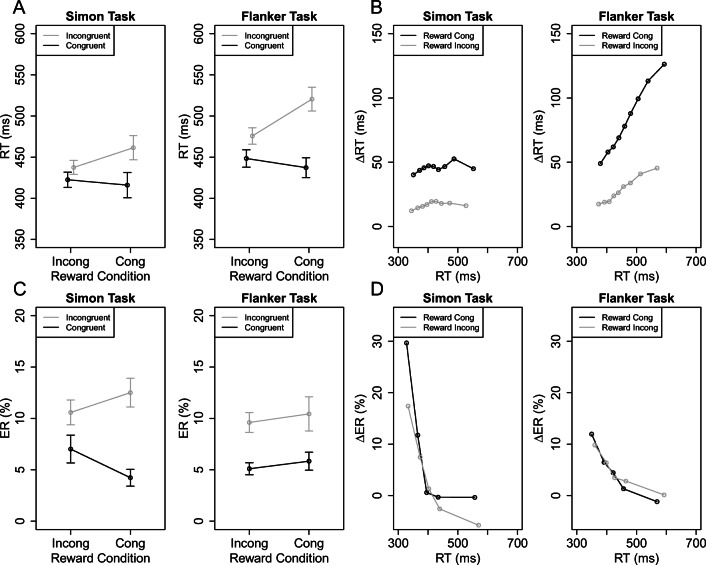
A. Mean reaction time (RT) as a function of reward (RC group, RI group) and congruency (congruent, incongruent) separately for the Simon and Eriksen flanker tasks in Experiment 2. B. Delta plots showing incongruent minus congruent differences in mean RT within each of 9 RT percentiles, plotted against the percentiles average RTs, separately for each task condition and participant. C. Mean percentage error (PE) as a function of reward group and congruency separately for the Simon and Eriksen tasks. D. Delta plots showing incongruent minus congruent differences in mean PE within each of 5 RT quantiles, plotted against the quantile mean RTs, separately for each task condition and participant. The error bars in A and C indicate 1 *SE* (standard error) of the corresponding means

Figure 3A shows the mean RTs as a function of reward (RC group, RI group) and congruency (congruent, incongruent) separately for the Simon and Eriksen flanker task. The 2 × 2 Simon-ANOVA revealed a significant main effect of congruency (449-419 = 30 ms), *F*(1, 56) = 38.12, *p* < .001, ${\eta ^{2}_{p}}$ = .40, which was further modulated by reward, *F*(1, 56) = 9.72, *p* = .003, ${\eta ^{2}_{p}}$ = .15. The Simon effect was larger in the RC than the RI group (45 ms versus 15 ms). The 2 × 2 Eriksen-ANOVA revealed also a significant main effect of congruency (498-442 = 57 ms), *F*(1, 56) = 77.60, *p* < .001, ${\eta ^{2}_{p}}$ = .58, and a significant interaction *F*(1, 56) = 19.84, *p* < .001, ${\eta ^{2}_{p}}$ = .26. The flanker effect was larger in the RC than the RI group (84 ms versus 28 ms). The overall 2 × 2 × 2 ANOVA revealed a significant main effect of congruency, *F*(1, 56) = 77.98, *p* < .001, ${\eta ^{2}_{p}}$ = .58, which was also further modulated by reward, *F*(1, 56) = 19.92, *p* < .001, ${\eta ^{2}_{p}}$ = .26. Furthermore, there was a significant main effect of task, *F*(1, 56) = 53.05, *p* < .001, ${\eta ^{2}_{p}}$ = .49, which interacted with congruency, *F*(1, 56) = 19.17, *p* < .001, ${\eta ^{2}_{p}}$ = .26. This two-way interaction basically indicated that the Eriksen flanker effect was generally larger than the Simon effect. Finally, there was a significant three-way interaction, *F*(1, 56) = 4.91, *p* = .031, ${\eta ^{2}_{p}}$ = .08. In sum, using a Simon-Eriksen-reward paradigm, we showed that rewarding incongruent versus congruent trials can not only reduce the mean Simon effect, but also the Eriksen flanker effect. This suggests that reward can modulate conflict processing with both location-based and flanker-based distracting information.^7^

Figure 3B shows the corresponding delta plots. The 2 × 2 ANOVA with the factors task and reward on mean slopes revealed significant main effects of task, *F*(1, 56) = 31.08, *p* < .001, ${\eta ^{2}_{p}}$ = .36, reward, *F*(1, 56) = 7.81, *p* < .001, ${\eta ^{2}_{p}}$ = .12, as well as a significant interaction *F*(1, 56) = 5.21, *p* = .026, ${\eta ^{2}_{p}}$ = .09. The slopes were generally less increasing in the RI than in the RC group for both Eriksen (0.40 versus 0.18) and Simon tasks (0.04 versus. 0.03), but the corresponding pairwise comparison revealed that this difference was only significant for the Eriksen but not the Simon task (with *p* = .001 and *p* = .802).

As can also be seen in Fig. 3B, the delta plots of the RI group were shifted downwards relative to the delta plots of the RC group for both conflict tasks. Exploratory analyses parallel to the one conducted in Experiment 1 confirmed this visual inspection. Specifically, there were significant differences when comparing the RC versus RI Simon effects (all *ps* < .003) and Eriksen flanker effects separately at each percentile (all *ps* < .001).^8^

#### Percentage errors (PEs)

Figure 3C shows the mean PEs as a function of reward and congruency separately for the Simon and Stroop task. The 2 × 2 Simon-ANOVA revealed a significant main effect of congruency, *F*(1, 56) = 37.16, *p* < .001, ${\eta ^{2}_{p}}$ = .40, and a significant interaction, *F*(1, 56) = 5.88, *p* = .019, ${\eta ^{2}_{p}}$ = .10. As for RTs, the Simon effect in error rates was larger for the RC than RI group (8.27% versus 3.56%). The 2 × 2 Eriksen-ANOVA revealed only a significant main effect of congruency (flanker effect of 4.55%), *F*(1, 56) = 30.79, *p* < .001, ${\eta ^{2}_{p}}$ = .35, (with all other *p* s > .549 and all other ${\eta ^{2}_{p}}$s < .01). Delta plots in error rates are visualized in Fig. 3D.

## General discussion

In the present study, we examined the influence of reward on conflict processing across three conflict tasks. Specifically, we compared Simon and Stroop effects (Experiment 1) and Simon and Eriksen flanker effects (Experiment 2) in groups that were selectively rewarded for congruent (RC) versus incongruent (RI) trials. Assuming that reward similarly affects conflict processing with different types of distractors, reward should generally strengthen congruent or incongruent distractor-response associations across all conflict tasks and this would increase (RC) or decrease (RI) the conflict effects (cf. Chen et al., 2020). In line with a distractor-general account, the mean Simon and flanker effects were larger in the RC than RI group. However, there was no evidence that the mean Stroop effect was modulated by reward, suggesting distractor processes differentially interact with target processes in the Stroop task as compared to the other tasks.

Of course, the present study does not imply that the reward manipulation may also modulate the Stroop effect under some circumstances (cf. Experiment 3 in Prével et al., 2021). Nevertheless, it is not clear how the different pattern could be reconciled without assuming distractor-specific processes since we controlled for methodological aspects as much as possible (e.g., using the same target dimensions and reward manipulations). Because all tasks have in common an overlap of distractor- and target-based activation produces conflict, it could be critical at which stage of processing these activations are superimposed. Considering that reward modulated both Eriksen flanker and Simon effects, the effects can be generalized across distractors producing both perceptual-related (Eriksen) and motor-related (Simon) informational conflict (cf. Hommel, 2011; Kornblum et al., 1999; Kornblum et al., 1990). Thus, it seems more likely that additional higher-level task conflict (i.e., reading the word and naming the color, cf.Goldfarb & Henik, 2007) prevents, or at least limits, control adjustments to distractor-specific reward contingencies.

In this context, it should be emphasized that there are at least two, mutually not exclusive, possibilities how reduced congruency effects in the RI compared to RC group could emerge (cf. Chen et al., 2020). First, reward could directly strengthen cognitive control by reinforcing the control states associated with incongruent (versus congruent) trials. Second, reward can indirectly modulate cognitive control by strengthening incongruent (versus congruent) distractor-response associations to predict responses. Hence, the conflict task-specific reward sensitivity in the present study could result from differences in the reinforcement of control association and/or reinforcement of distractor-response association.^9^

Thus, future studies are warranted to elucidate further how different types of distractor interfere with targets as a function of reward. Still, we think the present empirical similarities and differences across conflict tasks provides important additional insights that help to generalize further and constrain the influence of reward on cognitive control. Furthermore, it is also important that the development of more sophisticated theoretical accounts consider the effects of other reward manipulation (e.g., rewarding target processing). As reviewed in the introduction, there are some empirical discrepancies across previous conflict studies, and one might speculate that these distinct patterns could at least partially be due to the different types of distractors used (as opposed to the theoretically less interesting opportunity that these differences reflect methodological artifacts).

When theorizing about the effects of reward on conflict processing in this and in other studies, it is important to consider that the size of conflict effects depends on the interplay of the relative strength and *timing* of distractor-to-target processing. In this context, it seems useful to additionally examine the corresponding delta plot pattern. Previous studies have suggested that an offset of delta plots (i.e., shifts along the y-axis) primarily reflects the strength of suppressing distractor-based activation, whereas the slope of delta plots primarily reflects the timing of suppressing distractor-based activation (cf.Mittelstädt & Miller, 2020; Mittelstädt et al., 2022). The results suggest that in both the Simon and Eriksen flanker task the strength of suppressing distractor-based activation is adjusted by reward-contingencies since the delta plots of the RI group was shifted downward relative to the delta plot of the RC group. Interestingly, the results also demonstrated that, in particular, in the Eriksen task, the delta plot slope was significantly less strongly increasing in the RI compared to the RC group. Thus, rewarding of distractor-response association can modulate cognitive control not only via affecting the strength but also the time-course of suppressing distractor processing—at least under the presence of some types of distractors (flankers).

## Footnotes

1 Note, however, they observed a significant modulation when pooling the data of the first half of blocks of the two experiments

2 Note that (Prével et al., 2021) have also speculated that their different pattern across experiments may be due to methodological particularities. For example, they speculated that the adaptive RT threshold used in their first two experiments may have demotivated participants to adapt to adjust their behavior. Note, however, that (Chen et al., 2020) have observed strong effects with their adaptive RT thresholds.

3 Thus, the accuracy of both reward and non-reward trials were relevant for the overall number of points, but response speed was only relevant for reward trials. Note that the observed performance pattern between the RI and RC group in all experiment was generally quite consistent across error rates and RTs—that is, participants made, if any, more errors in the slower conditions.

4 Note that here and elsewhere, we always report the test-statistics using an uncorrected significance level of *α* = 0.05. Thus, one may also argue that this effect was not significant if adjusting the significance level of *α* = 0.05 to *α* = .025 for this additional exploratory analysis (Bonferroni-procedure).

5 As can be seen in the percentage error (PE) section, the mean Stroop PE effect was smaller than the mean Simon PE effect. While there was no evidence for a similar effect in RTs (i.e., congruency × task interaction, *F*(1, 57) = 0.33, *p* = .566, ${\eta ^{2}_{p}}$ = .01, it may still be possible that reward-specific modulations of Stroop effects could appear on a mean RT level if this effect is larger. To address the impact of the reward manipulation with overall larger congruency effects, we reanalyzed the data while only considering trials where the previous trial was congruent, because congruency effects are usually larger after congruent as compared to incongruent trials (e.g., Mittelstädt & Miller, 2018). However, the results were very similar to the reported ones—that, is the Simon RT effect was still significantly modulated by reward (*p* = .018), but not the Stroop effect (*p* = .762). More precisely, the Simon effect was larger in the RC than in the RI group (74 ms versus 53 ms), whereas the Stroop effect was descriptively even smaller in the RC than in the RI group (35 ms versus 41 ms). Thus, we do not think that the overall size of the Stroop effect is responsible for the lack of a reward-specific Stroop modulation.

6 As a further way to check whether the reward manipulation shifted the delta plots, we compared the RC versus RI intercepts of the fitted regression lines separately for each conflict task, adjusting the intercept to each individual mean RT (cf. Mittelstädt & Miller, 2020; 2018). In line with the exploratory analyses at each percentile, there was a significant difference for the Simon (*p* = .004), but not Stroop task (*p* = .856).

7 While the modulation is more pronounced with the latter distractor type in RTs, the reverse was true in error rates. Thus, the quantitative—not qualitiative—difference in size between reward-specific modulation of the Eriksen flanker and Simon effect in RTs may be (at least partially) due to differences in speed-accuracy tradeoffs (see e.g., Heitz, 2014; Liesefeld & Janczyk, 2019).

8 Further support for an offset of delta plots comes from significant differences of the RC versus RI delta plot adjusted intercepts (cf. Mittelstädt & Miller, 2020; 2018) for both the Simon task (*p* = .007) and Eriksen task (*p* < .001).

9 Note that (Chen et al., 2020) interpreted their results in terms of the second (distractor-response) association account, because their finding of *reversed* Simon effects in the RI group seem difficult to explain by accounts that assume that reward reinforces the implementation of control. However, because there were no reversed congruency effects on a mean or distributional level in the present experiments, our findings are compatible with both accounts.

## Declarations

All procedures performed in studies involving human participants were in accordance with the ethical standards of the institutional and/or national research committee and with the 1964 Helsinki Declaration and its later amendments or comparable ethical standards.
